# Single-cell atlas of diverse immune populations in the advanced biliary tract cancer microenvironment

**DOI:** 10.1038/s41698-022-00300-9

**Published:** 2022-08-18

**Authors:** Xuebing Shi, Zhixuan Li, Renqi Yao, Qingbao Cheng, Wei Li, Rui Wu, Zhihua Xie, Yanjing Zhu, Xinyao Qiu, Shuai Yang, Tao Zhou, Ji Hu, Yangqianwen Zhang, Tong Wu, Yan Zhao, Yani Zhang, Jianmin Wu, Hongyang Wang, Xiaoqing Jiang, Lei Chen

**Affiliations:** 1 Department I of Biliary Tract, Eastern Hepatobiliary Surgery Hospital, Naval Medical University, Shanghai, China; 2 The International Cooperation Laboratory on Signal Transduction, Eastern Hepatobiliary Surgery Hospital, Shanghai, China; 3 Translational Medicine Research Center, Medical Innovation Research Division and Fourth Medical Center of the Chinese PLA General Hospital, 100853 Beijing, China; 4 Department of Burn Surgery, the First Affiliated Hospital of Naval Medical University, 200433 Shanghai, China; 5 Department of Oncology, Fudan University Shanghai Cancer Center, Shanghai Medical College, Fudan University, Shanghai, China; 6 Institute of Metabolism and Integrative Biology and School of Life Sciences, Fudan University, Shanghai, China; 7 National Center for Liver Cancer, Shanghai, China; 8 Key Laboratory on Signaling Regulation and Targeting Therapy of Liver Cancer, Ministry of Education, Shanghai, China; 9 Shanghai Key Laboratory on Hepatobiliary Tumor Biology, Shanghai, China

**Keywords:** Cancer microenvironment, Immunoediting

## Abstract

Immunotherapies have been explored in treating solid tumors, albeit with disparate clinical effects in distinct cancer types. Systematic interrogation of immune cells in the tumor microenvironment (TME) is vital to the prediction of immunotherapy response and the development of innovative immunotherapeutics. To comprehensively characterize the immune microenvironment in advanced biliary tract cancer (BTC), we utilized single-cell RNA sequencing in unselected viable cells from 16 matched samples, and identified nineteen cell subsets from a total of 45,851 cells, in which exhausted CD8^+^ T cells, macrophages, and dendritic cells (DCs) in BTC were shown to augment and communicate within the TME. Transcriptional profiles coupled with T cell receptor (TCR) sequences revealed that exhausted CD8^+^ T cells retained clonal expansion and high proliferation in the TME, and some of them highly expressed the endoplasmic reticulum stress (ER) response gene, *XBP1*, indicating the role of ER stress in remodeling TME. Functional assays demonstrated that XBP1 and common immune checkpoints (PD1, TIGIT) were significantly upregulated in CD8^+^ T cells cocultured within the TME of BTC cells (GBC-SD, HCCC-9810). When treating the coculture groups with the specific inhibitor of IRE1α-XBP1 (4μ8C), the downregulation of TIGIT was observed in the treatment group. Collectively, comprehensive transcriptome profiling provides deep insights into the immune atlas in advanced BTC, which might be instrumental in exploring innovative immunotherapy strategies.

## Introduction

Biliary tract cancer (BTC) is composed of a group of tumors derived from anatomically different epithelial cells of the biliary tree, including intrahepatic cholangiocarcinoma (iCCA), extrahepatic cholangiocarcinoma (eCCA), and gallbladder carcinoma (GBC), which together account for approximately 1% of all adult cancers worldwide^1,2^. Over the past decade, it has become a major global concern due to its increasing morbidity and noticeable mortality^3,4^. However, compared with other common tumor types, BTC is still significantly understudied, and the progress of therapeutics has been limited in recent decades^5^.

During the last decade, checkpoint blockade therapies, CTLA-4 and PD-1 antibodies, have been a great success, especially in melanoma^6^, and they have also been applied in clinical trials for subsets of patients with advanced BTC^7,8^. However, pronounced clinical responses have been observed only in a fraction of patients. The observed discrepancy in treatment efficacy has been linked to the heterogeneity of infiltrating immune cells in the tumor microenvironment (TME)^9,10^. Recently, evolutionary single-cell transcriptome analysis has become a powerful technique for understanding cellular components and their heterogeneous phenotypic states residing in a highly complex TME at unprecedented resolution. This technique has been used to characterize immune cell populations in the TME of distinct cancers, including hepatocellular carcinoma (HCC)^11,12^, non-small-cell lung cancer^13^, melanoma^14^, and breast cancer^15^. These studies depicted high intratumor and interpatient heterogeneity in the immune spectrum, including prominent immunosuppressive subsets, newly defined cell populations, and communications crossing cell types, which have an essential influence on cancer progression. Recently, single-cell transcriptional analysis has been applied to unselected viable cells from iCCA patients, revealing that the intercellular crosstalk between malignant cells and vascular cancer-associated fibroblasts promotes tumor progression^16^. However, a comprehensive immune landscape of the TME in advanced BTC at single-cell resolution remains lacking.

In tumors, metastasis to drainage lymph nodes and other organs is a major cause of mortality. To corroborate the immune landscape of BTC in the process of tumor metastasis, we performed droplet-based 10X genomic single-cell RNA sequencing (scRNA-seq) on unselected viable cells isolated from five surgically removed BTC tumors and their matched peripheral blood samples, lymph node, or liver metastases (if available). In our study, we identified exhausted CD8^+^ T cells, DCs, and macrophages enriched in tumors with more frequent communications, indicating an immune-suppressed TME in advanced BTC. Strikingly, we found that an endoplasmic reticulum (ER) stress response gene, *XBP1*, was enriched in exhausted CD8^+^ T cells and functional assays revealed its role in the process of T cell dysfunction, which might help us to develop innovative immunotherapy strategies and predict clinical responses. Our results demonstrate the landscape of the immunosuppressive microenvironment in advanced BTC, which might provide a prospect for applying and exploring immunotherapies for BTC.

## Results

### Single-cell profiling of TME in advanced BTC

To better portray the complexity of immune cell populations in advanced biliary tract cancer, we undertook droplet-based 5′ scRNA-seq (10X Genomics) on unselected viable cells isolated from surgical tumor specimens, paired metastatic tissues and peripheral blood samples from 5 BTC patients. Selected patients including 2 iCCA, 2 GBC, and 1 distal cholangiocarcinoma (dCCA) were treatment naïve prior to tumor resection. Patients who met the inclusion criteria for scRNA-seq were identified by a specialist surgeon during the operation and immunohistochemical staining was performed after surgery. Detailed clinical and pathological information is provided in Table S1.

**Fig. 1:**
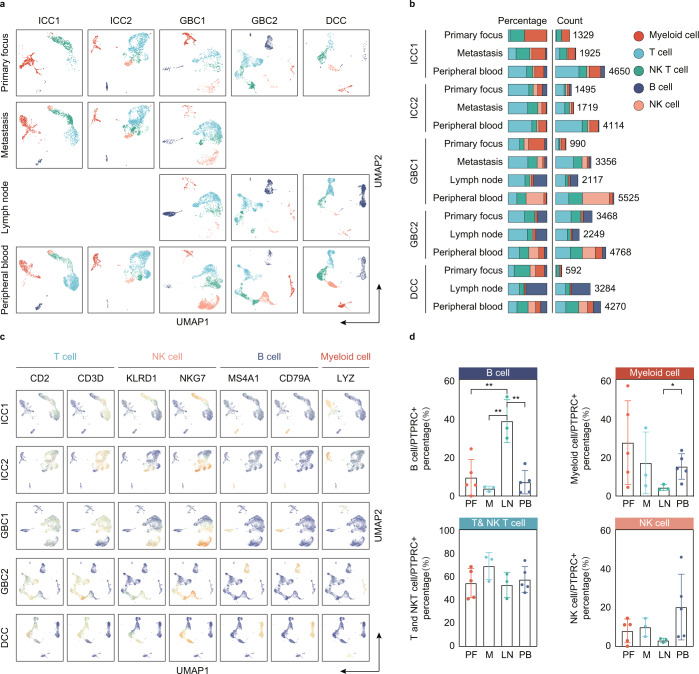
ScRNA-seq profiling of TME in advanced BTC. **a** The UMAP plot identified 4 main cell types in BTC, metastatic tissues and peripheral blood. **b** The number (right) and average proportion (left) of assigned cell types in different tissue types were presented. **c** UMAP plots of normalized marker expression of immune cells from all samples. **d** Bar plots depict percentage of B, T, NK, and myeloid cells in different tissues. Error bars indicate mean ± SEM. Two-tail paired *t* tests, **p < 0.01, *p ≤ 0.05.

To construct a global immune landscape, we filtered out CD45^-^ cells and a total of 45,851 CD45^+^ cells passing quality control were used for further analysis. On average, 67,192 uniquely mapped reads and 1277 genes per cell were profiled. As shown in Fig. 1a, four major immune cell types across disparate anatomic sites were verified in each patient and visualized using the uniform manifold approximation and projection (UMAP) algorithm (Fig. 1a, Table S3_ST.1)^17^. Based on expression of canonical genes, the identified immune cells included T cells (*CD2, CD3D*); NK cells (*KLRD1, NKG7*); B cells (*MS4A1, CD79A*) and myeloid cells (*LYZ*) (Fig. 1c, Table S3_ST.3). All these cell populations were shared among patients and between distinct tissues with different proportions and cell counts (Fig. 1b, Table S3_ST.2), revealing substantial inter-and intra-patient heterogeneity of immune cell compositions among BTCs. In agreement with other studies^18^, we found that the T-cell lineage was the most prevalent immune cell type in all tissues (Fig. 1d). Meanwhile, we observed that the proportion of B cells isolated from lymph nodes was much higher than those in tumor and peripheral blood (Fig. 1d), which was consistent with the finding of Elham et al.^15^, whereas myeloid cells in lymph node were significantly lower, indicating the immune microenvironment of tumor draining lymph node is not negatively regulated by myeloid cells, which may represent a completely different tumor microenvironment from primary or other metastatic foci (Fig. 1d).

### Clustering and subtype analyses of T, B, NK, and myeloid cells

**Fig. 2:**
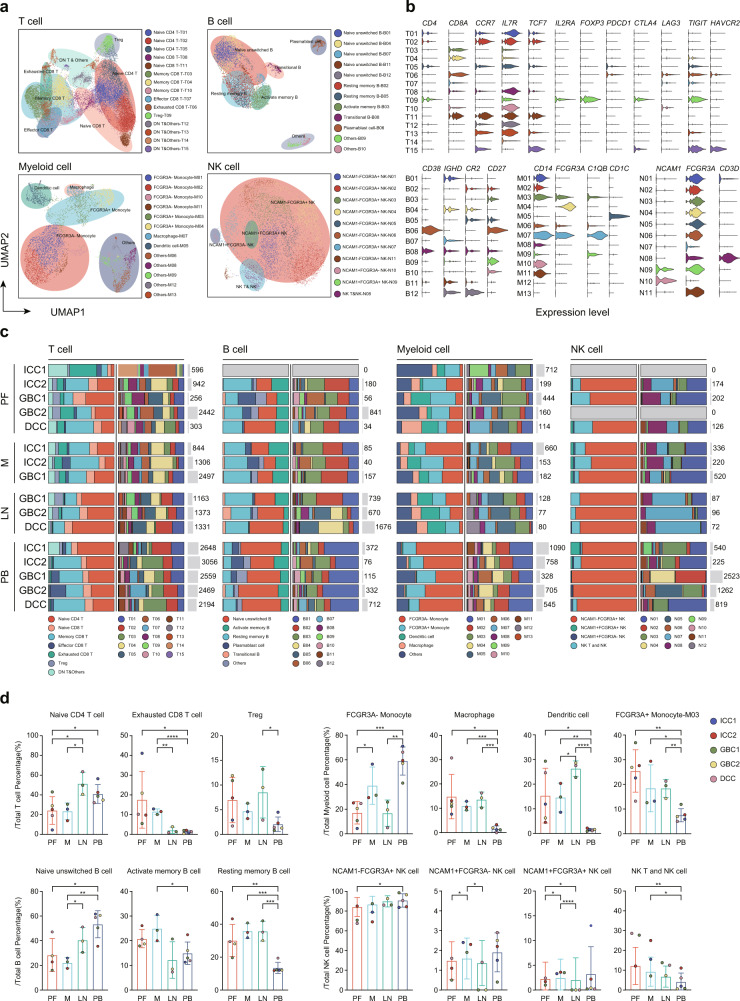
Clustering and subtype analyses of T, B, NK and myeloid cells. **a** UMAP plot of subclustered T, B, NK, and myeloid cells, labeled in different colors. Cell type annotations are provided in the figure. **b** Violin plots showing marker genes for distinct immune cell subtypes. **c** The number (right) and average proportion (left) of assigned cell subsets in different tissue types were presented. **d** Bar plots depict percentage of subsets of B, T, NK, and myeloid cells in different tissues. Error bars indicate mean ± SEM. Two-tail paired *t* tests, ****p < 0.0001, ***p < 0.001, **p < 0.01, *p ≤ 0.05.

**Fig. 3:**
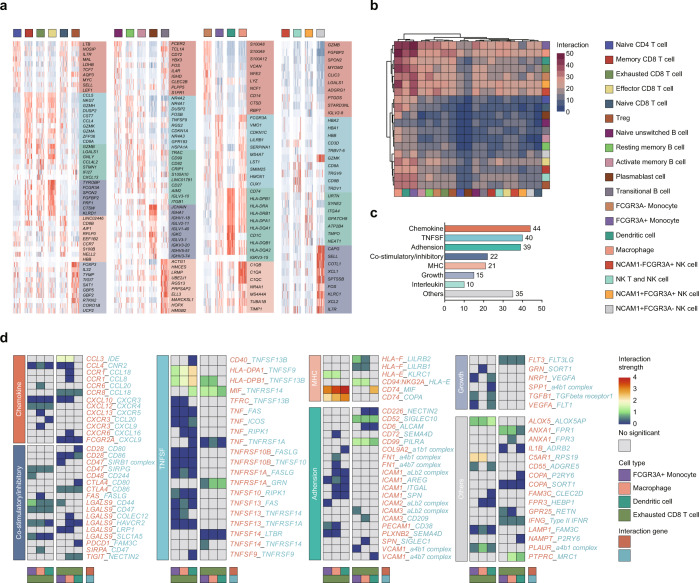
Cell–cell interactions between myeloid cells and exhausted CD8^+^ T cells. **a** Heatmap expression showing the top 10 DEGs (Wilcoxon test) in each cluster. **b** Heatmap of the number of significant ligand-receptor interactions between all immune cell types, demonstrating a substantial number of interactions between myeloid cells (*FCGR3A*^*+*^ monocyte, DCs, macrophage) and exhausted CD8^+^ T cells. **c** A reference list of significant interactions between myeloid cells and exhausted CD8^+^ T cells. **d** Heatmap expression showing significant ligand-receptor pairs between myeloid cells and exhausted CD8^+^ T cells.

Given that immune cells have a major influence on oncogenesis and progression^19,20^, T, B, NK and myeloid cells were further partitioned through reclustering analyses and yielded 11, 10, 11, and 8 clusters, respectively (Fig. 2a, Table S3_ST.4). These clusters were further categorized and annotated by canonical marker genes (Fig. 2b) as well as the enrichment of differentially expressed genes (DEGs) (Fig. 3a). For B cells, five kinds of cell types were obtained, including naïve unswitched B cells (characterized by the expression of *IGHD* and *CR2*), activated memory B cells, resting memory B cells, plasma blast cells and transitional B cells (Fig. 2a). The distribution of B cells was comparable among patients (Fig. 2c, Table S3_ST.5). For example, naïve unswitched B cells were prevalent in peripheral blood and lymph nodes. In contrast, resting memory B cells increased significantly in tumors and metastases (Fig. 2d). Likewise, by using known functional NK cell markers, we identified four populations, including *NCAM1*^*-*^*FCGR3A*^*+*^ NK cells, *NCAM1*^*+*^*FCGR3A*^*+*^ NK cells, *NCAM1*^*+*^*FCGR3A*^*-*^ NK cells, and NK T cells (Fig. 2a). Of which, *NCAM1*^*-*^*FCGR3A*^*+*^ NK cells accounted for the majority (~85%) of all tissues, whereas *NCAM1*^*+*^*FCGR3A*^*-*^ NK cells and *NCAM1*^*+*^*FCGR3A*^*+*^ NK cells were less than 5%. The number of NK T cells in the tumor and metastatic foci was significantly higher than in peripheral blood (Fig. 2d), suggesting their complex antitumor effect in the TME^21^. Additionally, we confirmed the diversity of myeloid cells, and *FCGR3A*^*-*^ monocyte, *FCGR3A*^*+*^ monocyte, macrophage, and dendritic cell subsets were identified (Fig. 2a). The composition of myeloid cells was distinct from that of their primary, metastatic tissues and peripheral blood counterparts (Fig. 2c). For example, unique distributions of the *FCGR3A*^*-*^ monocyte subset were enriched in the peripheral blood, accounting for 50%–70% of the total percentage (Fig. 2d). In contrast, we observed significantly increased DCs, macrophages and *FCGR3A*^*+*^ monocytes in primary foci, metastatic tissues (Fig. 2d), suggesting their regulatory role in the TME. We found that macrophages in our study preferentially expressed *C1Qs* (Fig. 3a). As suggested in colorectal cancer, *C1Q*^*+*^ macrophages tend to be dominant in tumors and play roles in recruiting and regulating regulatory CD4^+^ T cells (Tregs) and exhausted CD8^+^ T cells, implying the immunosuppressive function of *C1Qs*^*+*^ macrophages^22,23^.

Tumor-infiltrating T cells are highly heterogeneous and have been shown to play crucial roles in immune evasion and immunotherapy response. To gain insight into the intrinsic structure and potential functional states of overall T cell populations, we performed unsupervised clustering of T cells and a total of 11 stable clusters emerged, including three clusters for naïve CD4^+^ T cells (T01, T02, T05), two for naïve CD8^+^ T cells (T08, T11), three for memory CD8^+^ T cells (T03, T04, T10), one for effector CD8^+^ T cells (T07), exhausted CD8^+^ T cells (T06) and Treg (T09) (Fig. 2a). Naïve CD4^+^ T cells were dominant in peripheral blood and lymph nodes (Fig. 2d) and specifically expressed “naïve” marker genes such as *TCF7*, *IL7R* and *SELL* (Fig. 3a, Table S3_ST.6), representing immature T cells^24^. Of interest, memory, effector, and exhausted CD8^+^ T cells shared differentially expressed genes, containing a subset of cytotoxicity-associated genes including granzymes and perforins (*GZMB* and *PRF1*) (Fig. 3a), suggesting an activated effector state. In addition to the gene characteristics of the effector program in these cells, exhausted CD8^+^ T cells alternatively expressed remarkably well-described exhausted genes such as *CXCL13* (Fig. 3a)^12,25^, which suggested that these cells respond reactively to tumor antigens and transformed into different subsets and immune states. Notably, we observed fewer effector T cells but more memory and exhausted T phenotypes in the TME (Fig. 2c). Recent studies^26,27^ from the Held and Reiner groups demonstrated that *TCF1*^*+*^/*TCF7*^*+*^ stem-like CD8^+^ T cells early after activation differentiate into a small number of effector cells and a large number of memory T cells^28^. Moreover, several studies^12–14^ have reported that GZMK^+^CD8^+^ T cell defined as a pre-dysfunctional or transitional state could transform into an exhausted state under persistent tumor antigen stimulation. Interestingly, in our study, naïve CD8^+^ T cells highly expressed *TCF7*, and *GZMK* was differentially enriched in memory CD8^+^ T cells (Fig. 3a). Consequently, we speculate that naïve CD8^+^ T cells may differentiate into different functional states under the stimulation of tumor antigen^29^. Moreover, Núñez and colleagues^30^ observed that Treg frequencies increase with metastatic lymph nodes of breast cancer, and a common transcriptomic signature, *CD80*, in Tregs is significantly associated with poor survival, which is more aligned with our findings (Figs. 2c, d, 5a). Collectively, we obtained diverse immune cell phenotypes, especially immunosuppressive cells in the TME at single-cell resolution.

### Cell-cell interactions between myeloid cells and exhausted CD8^+^ T cells form an immunosuppressive milieu

Cell-cell communications, mainly through ligand-receptor (L–R) interactions, play unique roles in reprogramming the tumor microenvironment and response to therapeutics^31^. Hence, we quantified the potential cell-cell communications across all immune cell types using the CellPhoneDB package^32^ with a reference list of literature-supported interactions including L–R pairs from chemokines, tumor necrosis factor super family (TNFSF), costimulatory/inhibitory, adhension, major histocompatibility complex (MHC), growth, and others (Fig. 3b, Table S3_ST.7), and found more frequent interactions between myeloid cells (*FCGR3A*^*+*^monocyte, macrophage, DCs) and exhausted CD8^+^ T cell (Fig. 3b), which is consistent with the vital roles of myeloid cells as immune regulators^33,34^. Most of the highest-scoring interactions were part of the chemokine family (Fig. 3c), implying that these immunosuppressive cells may transmit across tissues through chemokine L-R pairs. Zhang et al.^35^ found that CXCL10 secreted by M2 macrophages interacts with CXCR3 on Tregs to recruit Tregs and induces an immunosuppressive microenvironment. Moreover, we also observed substantial costimulatory/inhibitory receptors between exhausted CD8^+^ T cells and myeloid cells, including CD47-SIRPG, TIGIT-NECTIN2, and CTLA4-CD86 interactions. As found by Ho’s work^36^, macrophages suppressed tumor-infiltrating lymphocytes (TILs) and, through TIGIT-NECTIN2 interaction with complementary T cells, shaped an immunosuppressive environment in HBV-associated hepatocellular carcinoma. Collectively, frequent crosstalk between exhausted T cells and myeloid cells may play an important role in promoting immune escape, and targeting these receptors for immunotherapy may help to restore the antitumor immune response in advanced BTC^37^.

### Clonal enrichment of exhausted CD8^+^ T cells in the TME revealed by TCRs

**Fig. 4:**
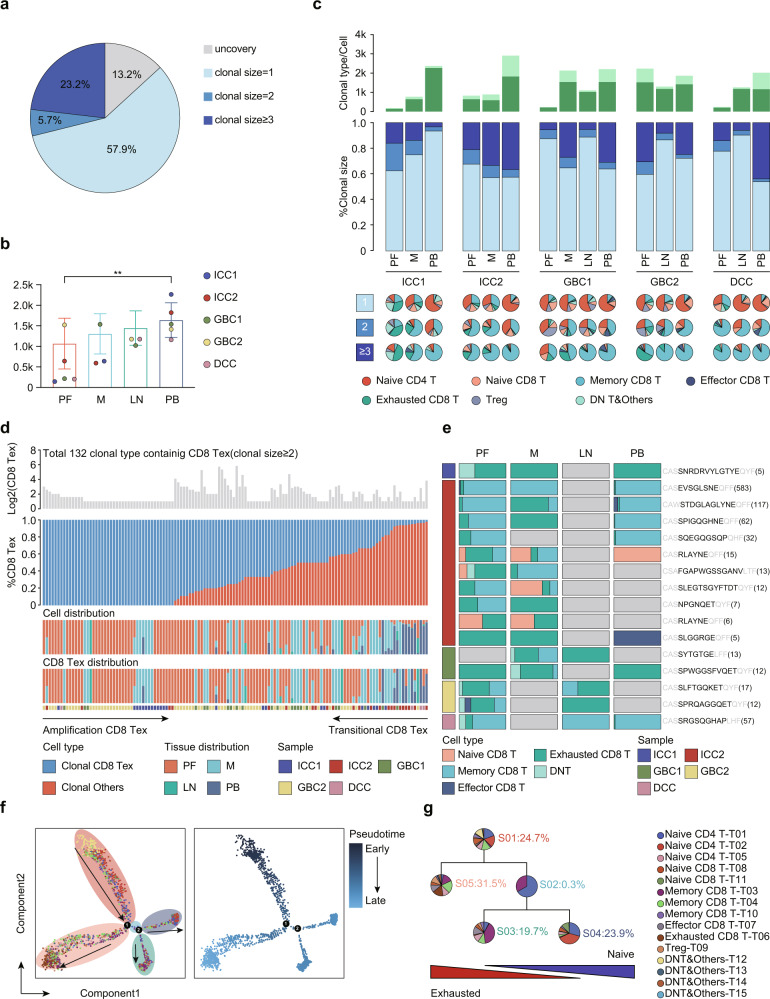
Clonal enrichment of exhausted CD8^+^ T cells in the TME revealed by TCRs. **a** pie charts illustrate the composition of every individual TCR. **b** Bar plots depict the number of clone types in different tissues. **c** Clonal compositions of T cell in different tissues across patients, showing from top to bottom the number/types of clone, the distribution of clones by size (size = 1, size = 2, size ≥3 cells), and Pie charts illustrating the composition of subsets of T cell stratified by clone size. **d** Clonal types of exhausted CD8^+^ T cell in different tissues across patients, showing from top to bottom the number of exhausted CD8^+^ T cells corresponding to each clone type, the ratio between exhausted CD8^+^ T cell and transitional CD8^+^ T cells, the cell and exhausted CD8^+^ T cells distribution among different tissues across individual patients. **e** distribution of distinct T cell subtypes in different tissues with the same TCR β alleles (black font). **f** Monocle 2 trajectory analysis of T cells annotated by cell subgroups (left panel) and pseudotime (right panel). **g** The pie chart showing the fraction of T cell compositions at different states of pseudotime trajectory analysis. Arrows show the increasing directions of certain T cell properties. Error bars indicate mean ± SEM. Two-tail paired *t* tests, **p < 0.01.

Because TCRs often serve as specific identifiers of T cell ancestries^38^, we used our scRNA-seq data to trace the lineage of each single T cell according to their full-length TCR. We obtained TCRs with α and β chains for 21,879 cells and 21,224 cells, respectively, of which 57.9% contained unique TCRs (clonal size = 1) and 28.9% harbored repeated use of TCRs (clonal size ≥ 2), indicative of clonal expansion (Fig. 4a), which is compatible with previous studies that most cells expressed unique TCR α and β alleles^12,18^. We found that T cell infiltrations in peripheral blood showed a diverse TCR spectrum with minimal clonal expansion, while those in tumor lesions were strongly dominated by oligoclonal expansion of specific T cell phenotypes, especially more abundant clonally exhausted CD8^+^ T cells (Fig. 4b, c). In light of the low clonal enrichment of exhausted CD8^+^ T cells in blood, the clonal accumulation of exhausted CD8^+^ T cells was probably the result of local activation and proliferation in the tumor environment, as suggested by previous reports^12,39^. In addition, memory CD8^+^ T cells displayed increased clonal expansion across all tissues, especially in blood and lymph nodes (Fig. 4c), implying that the infiltration of these cells into tumors may be a mechanism that provides the external sources of exhausted CD8^+^ T cells in tumors in addition to local expansion^23^. Currently, a pivotal role of memory CD8^+^ T cells in providing antitumor function has become more appreciated. Clonotypic expansion of memory T cells in our study may represent a kind of precursor cell of TILs that might be replenished in tumor sites following immunotherapy^40,41^.

We further analyzed and obtained a total of 132 clone types across exhausted CD8^+^ T cells (Fig. 4d). The induction of the exhaustion subsets of these tumor-infiltrating CD8^+^ T cells may be due to long-term exposure to their respective antigens. Interestingly, approximately 1/3 of the exhausted CD8^+^ T cell clone types showed monoclonal expansion in the TME, while the others showed shared TCRs between transitional CD8^+^ T cells and exhausted CD8^+^ T cells, indicating ongoing transitional CD8^+^ T cell differentiation toward exhausted CD8^+^ T cells (Fig. 4d). When comparing cell distribution with exhausted CD8^+^ T cell distribution, we observed noticeable shared TCRs among peripheral blood and tumor lesions and more exhausted CD8^+^ T cells enriched in the tumor loci by decreasing in peripheral blood (Fig. 4d), indicating that a fraction of exhausted CD8^+^ T cells are derived from circulating blood. Combined with TCR analysis, these results confirm our previous speculation that exhausted CD8^+^ T cells can not only be cloned and expanded locally, but also can be transformed from other T cell types, and even from peripheral blood-derived T cell types. Similarly, we found that distinct T cell types in different tissues represent shared TCR β alleles (Fig. 4e), suggesting that these cells may target the same antigen but undergo disparate state transitions in different tissue environments. To understand the links between distinct T cell subsets, we carried out an unsupervised inference method, Monocle^42^, to construct the potential developmental trajectories of T cell clusters. The inferred developmental trajectory yielded a branched structure, in which exhausted T cells were positioned at the opposite end of naïve clusters (Fig. 4f, g, Table S3_ST.8). These exhausted T cells were highly enriched in the late pseudotime period (Fig. 4f, g, Table S3_ST.9). Collectively, coupled TCR and trajectory analysis suggested that, during the chronic stimulation of cognate antigen, T cells gradually activated and transformed into a late dysfunction state and clonally expanded in situ^43^.

### A proliferative state in dysfunctional T cells

**Fig. 5:**
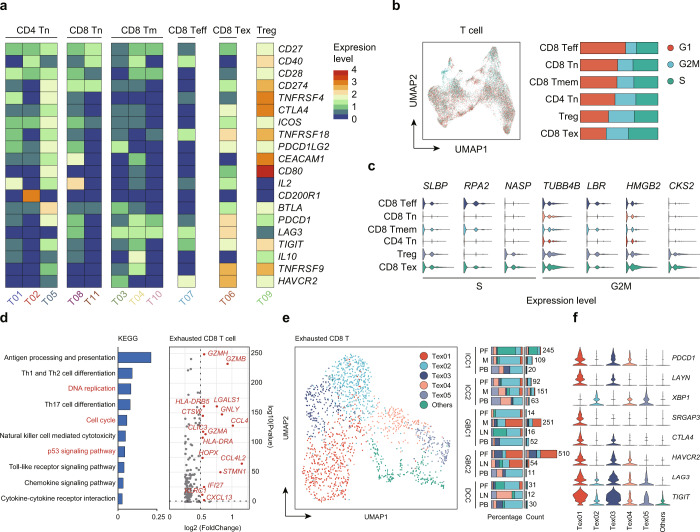
A proliferative state in dysfunctional T cells and subtype analyses of exhausted CD8^+^ T cells. **a** Heatmap of the expression patterns of genes currently targeted by immunotherapies. The color density indicates the average expression of a given gene, with each row normalized by z-score. **b** UMAP plot of distinct cell cycles among T cells, labeled in different colors (left). The average proportion of assigned cell cycles in different T cell subgroups were presented (right). **c** Violin plots of proliferation genes in distinct T cell subtypes. **d** Volcano plot showing differentially expressed genes in tumor-infiltrating exhausted CD8^+^ T cells. Each red dot denotes an individual gene passing our *p* value and fold difference thresholds (right). KEGG analysis of DEGs in exhausted CD8^+^ T cells (left). **e** UMAP plot for 6 distinct exhausted CD8^+^ T cells. The number (right) and average proportion (left) of assigned cell types in different tissue types were presented. **f** Violin plots showing marker genes for five distinct exhausted CD8^+^ T cell subtypes.

Among T cells, costimulatory receptors, such as CD28, ICOS, and CD40, markedly enhance TCR-dependent T cell activation, whereas high levels of the inhibitory receptors CTLA-4, PDCD-1, and TIGIT are signatures of progressive T cell exhaustion^44,45^. These receptors could be pursued as cancer immunotherapy targets in current clinical trials^46^. Interestingly, some of these genes activated in Tregs overlapped with those features of exhausted CD8^+^ T cells (e.g., *TNFRSF9*, and *TIGIT*). In contrast, *CTLA4* and *CD80* were enriched only in Tregs, while *PDCD1* and *LAG3* were preferentially enriched in exhausted CD8^+^ T cells (Fig. 5a, Table S3_ST.10, ST.11), implying a highly activated and dysfunctional state in exhausted CD8^+^ T cells and Tregs (Fig. 5a). Furthermore, we generated a proliferation score for each cell by pooling the expression level of cell cycle-related genes (*TUBB4B, HMGB2*)^47,48^. The highest proportion of proliferative cells was observed in exhausted CD8^+^ T cells (Fig. 5b, c). In addition, compared to naïve-like or cytotoxic T cells, Treg populations also showed a higher proportion of cells expressing proliferation-associated genes (Fig. 5b, c). Furthermore, we explored DEGs in exhausted CD8^+^ T cells, including *CXCL13*, and *STMN1*, etc. (Fig. 5d). KEGG analysis of these DEGs suggested that these cells were exposed to varying degrees of DNA replication, cell cycle, and p53 signaling pathway (Fig. 5d), indicating their proliferative states. As such, cell cycle analysis, transcriptional profiling and TCR revealed that although exhausted CD8^+^T cells and Tregs clonally expanded and highly proliferated in situ, these cells had developed varying degrees of functional impairment, which might be the major reason for immunosuppression in the TME and indicate their potential ability to respond to immunotherapy^49^.

### TME induced XBP1 and other classical immune checkpoints (PD1, TIGIT) in CD8^+^ T cells

**Fig. 6:**
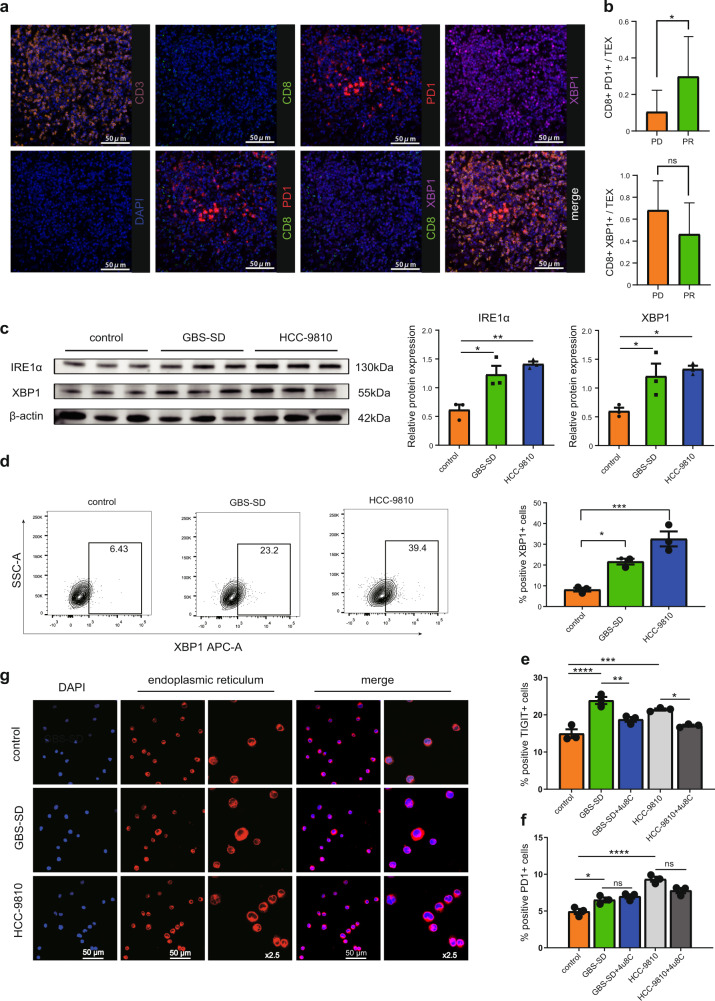
Functional assays for exhausted CD8^+^ T cells. **a** Detection of PD1^+^CD8^+^ T cells and XBP1^+^CD8^+^ T cells in BTC tissue by multiplex immunohistochemistry staining. Representative data from eighteen patients were shown. **b** Quantitation of XPB1^+^CD8^+^ T cells and PD1^+^CD8^+^ T cells as a percentage of total exhausted CD8^+^ T cells in two different response groups. **c**–**f** CD8^+^ T cells were stimulated with anti-CD3 and anti-CD28 for 96 h and cultured with the supernatant from HIBEpiC, HCCC-9810 and GBC-SD for 48 h. **c** the protein level of both IRE1α and XBP1in cocultured CD8^+^ T cells tested by western blotting. β-actin were used as loading control. (representative plots on left; quantitation on right). **d** Representative FASC plots gated on XBP1^+^ cells, with positive cells (XBP1^+^CD8^+^) boxed (left). Shown is the quantitation of positive cells as a percentage of total CD8^+^ T cells (right). **e**, **f**. 10 µM 4μ8C was added to the above medium for 24 h before harvesting. Quantitation of FACS plots of positive TIGIT^+^ T cells (**e**) and PD1^+^ T cells (**f**) as a percentage of total CD8^+^ T cells. **g** CD8^+^ T cells were stained with ER tracker (red) and nuclei were stained using DAPI (blue). Morphologies of endoplasmic reticulum were analyzed by laser scanning confocal microscopy from control, GBS-SD, and HCCC-9810 cell lines. Error bars indicate mean ± SEM. Two-tail paired *t* tests, ****p < 0.0001, ***p < 0.001, **p < 0.01, *p ≤ 0.05, no significance.

To further address the intrinsic exhausted CD8^+^ T cell heterogeneity, we applied unsupervised clustering based on UMAP and identified five exhausted CD8^+^ T cell subsets (Fig. 5e, Table S3_ST.12, ST.13). Exhausted marker genes exhibited distinct expression patterns among our corresponding five exhausted CD8^+^ T cell clusters: Tex01, Tex02, Tex03, Tex04, and Tex05, although *LAG3* and *TIGIT* were uniformly highly expressed among exhausted CD8^+^ T cell subtypes. For example, Tex01 and Tex03 cells expressed high levels of *PDCD1, LAYN, CTLA4*, and *HAVCR2* but low levels of *XBP1*, whereas Tex02 and Tex05 cells exhibited the opposite pattern (Fig. 5f, Table S3_ST.14). Notably, a higher number of coinhibitory receptors within the Tex01 and Tex02 clusters may represent more severe exhaustion^44^. The mutually exclusive expression pattern of *PDCD1* and *XBP1* suggests that *XBP1* might serve as a marker for a different subset of exhausted CD8^+^ T cells. Accordingly, we roughly divided exhausted CD8^+^ T cells into two groups: the preferential *PDCD1* enrichment group and the typical *XBP1* expression group. To this end, we hypothesize that multiplex immunohistochemistry (mIHC) staining based on CD3, CD8, PD1, and XBP1 antibodies may validate the distinction and the quantity of the two exhausted CD8^+^ T cell groups at the protein level in an additional 18 iCCA patients who received anti-PD-1 therapy and had available clinical response data evaluated by RECIST criteria (*n* = 9, partial response (PR)/complete response (CR); *n* = 9, progressive disease (PD); talbleS2)^50^. As expected, representative mIHC images confirmed varying expression levels of PD1^+^CD8^+^ T cells and XBP1^+^CD8^+^ T cells in these iCCA tumor tissues (Fig. 6a). Strikingly, by interrogating the clinical response with respect to the effects of different exhaustion subsets, we noticed that in the response group, a higher level of PD1^+^CD8^+^ T cell infiltrates was observed, while the proportion of XBP1^+^CD8^+^ T cells was lower (Fig. 6b). Therefore, it is plausible that the compartment of such cell types might allow effective patient stratification, and distinct subgroups of patients may need to adopt different immunotherapy strategies.

To corroborate whether the BTC tumor microenvironment can induce the upregulation of common checkpoint receptors and ER stress in CD8^+^ T cells, we treated CD8^+^ T cells isolated from the peripheral blood of healthy people with conditioned media collected from two human BTC cell lines (GBC-SD, HCCC-9810) and a normal bile duct cell line (HIBEpiC, as a control). Intriguingly, CD8^+^ T cells cultured in conditional medium from GBC-SD and HCCC-9810 showed significantly potentiated IRE1α-XBP1 pathway in comparison with that of CD8^+^ T cultured in HIBEpiC medium, as evidenced by increased expression level of both IRE1α and XBP1 using western blot analysis (Fig. 6c). In addition, using flow cytometry analysis, the expression of XBP1 (Fig. 6d) and checkpoint receptors (TIGIT, PD1) (Fig. 6e, f) in cocultured CD8^+^ T cells from BTC cells was parallelly upregulated, accompanied with overactivated ER stress, as evidenced by the enlargement of the ER via confocal laser scanning microscopy (Fig. 6g). As expected, when treating the coculture groups with the specific inhibitor of IRE1α-XBP1 (4μ8C), we observed the downregulation of TIGIT in the treatment group, suggesting that XBP1 might serve as an underlying target for innovative antitumor therapy (Fig. 6e). Intriguingly, inhibiting XBP1 did not reverse the expression of PD1 (Fig. 6f). Our transcriptome and functional analysis show that there are two main types of exhausted CD8^+^ T cells in the TME, one of which is a group of generally defined exhausted T cells expressing substantial checkpoint receptors, and the other is a mass of dysfunctional T cells that specifically express XBP1 due to ER stress. In turn, the mechanism of ER stress in immune cell dysfunction and its potential value in immunotherapy deserve further effort.

## Discussion

Despite the durable response in cancer immunotherapy, the roadblock of understanding the mechanisms of the immune response or predicting efficacy remains the heterogeneous components of immune cells within tumors. Several studies have conducted scRNA-seq to assess the heterogeneity of the tumor microenvironment across a variety of malignancies^11,16,18^. These studies provided deeper insights into a better understanding of the immunosuppressive tumor microenvironment. Using unbiased scRNA-seq analysis, we constructed an immune atlas in advanced biliary tract cancers and unveiled vast versatile immune cells from both the innate and adaptive immune systems, especially in the TME. Of interest, we observed that NK cells absented in the primary focus of iCCA1 and GBC2 patient, accompanied by remarkable clonally expanded (clonal size ≥ 3) exhausted CD8^+^ T cells (Figs. 1b, 2c, 4c), which may indicate a longer course of disease and an advanced stage of illness^12^. Comparing the pathological data of iCCA1 with iCCA2, albeit they were in the same stage of Tumor-Node-Metastasis (TNM), we found that iCCA1 developed diffuse peritoneal metastasis, while iCCA2 had locally resectable intrahepatic metastasis. Similarly, when comparing GBC2 with GBC1, we found that GBC2 developed more lymph node metastases (3 vs. 1 nodes), indicating that the value of immunophenotype in patient stratification may be more sensitive than that of the traditional pathological staging^51,52^. In addition, we found that, compared with iCCA or dCCA, more Tregs with clonal expansion (clonal size = 2) were observed in primary lesions of GBC (Fig. 4c). Considering that the overall survival rate (OS) of GBC patients is worse than that of other anatomical BTC patients^53^, whether there is a correlation between the two needs further research. It is noteworthy that the arguments were derived from our inadequate data, and a more robust conclusion needs to be confirmed with analyses of large samples and investigations of the potential mechanism.

Since exhausted CD8^+^ T cells and Tregs preferentially enriched in primary tumors and metastatic foci may serve as potential clinical targets, we characterized T cells through transcriptome and TCR analysis and found that dysfunctional T cells clonally expanded and highly proliferated in situ. Notably, this characteristic seems to be shared among different tumor types^14,18^. By reference, proliferative exhausted CD8^+^ T cells in advanced BTC may respond to immune checkpoint inhibitors and be characterized as potential immunotherapy targets^54,55^. With this strategy, we further focused on and explored exhausted CD8^+^ T cells. Interestingly, we found that ER stress response gene, *XBP1*, was dramatically enriched in a group of exhausted CD8^+^ T cells, which was different from conventional exhausted CD8^+^ T cells expressing a number of coinhibitory receptors. To identify the varying expression levels of these two exhausted CD8^+^ T cell types, we used multiplex immunohistochemistry method of marker genes (CD3, CD8, PDCD1, XBP1) in paraffin slices from 18 iCCA patients who were treated with anti-PD1 therapy. Interestingly, we noticed that a high ratio of *PD1*^*+*^ CD8^+^ T cells to *XBP1*^*+*^ CD8^+^ T cells was associated with better response to anti-PD-1 therapy, which indicates that it might be used as a biomarker to predict the response to anti-PD-1 therapies^56–58^. Notably, several studies have shown that *XBP1*, an ER stress response gene, plays an important role in T cell exhaustion, and suggest a new strategy for activating T cell cytotoxicity by targeting ER stress to enhance T cell-based immunotherapy^59–61^. However, the role of ER stress in biliary tract cancer is still unclear. Consistent with expectations, the upregulation of inhibitory receptors (PD1, TIGIT) and XBP1 was observed when coculturing CD8^+^ T cells with the supernatant of BTC cells, suggesting that ER stress and exhausted CD8^+^ T cells were induced and formed in the tumor microenvironment. In turn, a small molecular inhibitor (4μ8C) targeting IRE1α-XBP1 signaling could significantly downregulate the expression of the coinhibitory receptor TIGIT. Johnston and colleagues^62^ observed that the deletion of TIGIT was able to enhance both CD4^+^ and CD8^+^ T cell effector functions. As inference, the exhaustion process may be potentially abrogated when treated with XBP1 inhibitors, and manipulating XBP1/changing TILs metabolism may improve the clinical response and prolong the prognosis of BTC patients^59,63,64^. Clinically, statins, cholesterol-lowering drugs that represent a mechanism to manipulate ER stress, have been reported to reduce cancer-related mortality^65^. A recent study from the Roberts group reported that statins improve survival in patients with dCCA^66^. Intriguingly, XBP1 inhibitor did not reverse the expression of PD-1 in our assays, which may consist with the work from Song et al.^61^ that targeting XBP1 is likely to be independent of common immune checkpoint (PD1, CTLA-4, or TIM-3) signaling. In general, it is plausible that controlling ER stress or inhibiting XBP1 may be a potential prospective immunotherapy strategy for BTC patients.

ScRNA-seq has been applied to explore the tumor microenvironment of a variety of cancer types, while the research on biliary tract cancer is still insufficient. Of which, Liu et al.^35^ analyzed the difference between ErbB pathway mutation and non-mutation group within 13 patients with gallbladder cancer, and found that in the mutation group, the higher levels of midkine (MDK) secreted by epithelial cells interacted with its receptor LRP1 expressed by macrophages to promote immunosuppressive microenvironment. Likewise, Chen et al.^67^ revealed the heterogeneity and interactions of cells in gallbladder cancer, indicating its potential role in tumor progression. In our study, we used single-cell RNA and TCR sequencing on CD45^+^ immune cells, including their heterogeneity, dynamics, and potential functions. Consistent with previous studies, a group of exhausted CD8^+^ T cells expressing substantial exhaustion genes with remarkable clonal expansion were observed in the immunosuppressive TME. Specifically, a group of less described exhausted CD8^+^ T cells highly expressing *XBP1* were observed in the TME, suggesting the potential role of ER stress in the process of T cell dysfunction, and the potential opportunity of developing innovative immunotherapeutics.

Notably, considering the accessibility of specimens from patients with advanced diseases, we pooled the three subtypes of BTC by necessity to obtain adequate sample size, which inevitably introduced bias. In addition, compared with previous studies^11,13^, we found that the number of TILs in our study was slightly small. Therefore, we failed to obtain vast diversity subtypes, such as pre-exhausted CD8^+^ T cells or highly exhausted CD8^+^ T cells. We hypothesize that fluorescence-activated cell sorting (FACS) before constructing the library may augment the number of target cells.

In summary, our transcriptional map of immune cells from advanced BTC provided a framework for understanding the various immune status and portrayed the dynamic characteristics of immune cells in the BTC setting. Coupled with TCR sequencing, we identified highly proliferative and clonally expanded dysfunctional T cells in the tumor microenvironment, indicating an immune-suppressive state and potential targets for developing immunotherapies in BTC. Finally, we observed a less described dysfunctional marker, *XBP1* (an ER stress response gene), enriched in exhausted CD8^+^ T cells, which may help us to stratify patients and evaluate therapeutic efficacy. Collectively, our results revealed an atlas of suppressive immune cells in the TME and set the horizon for the application and development of immunotherapies for biliary tract cancer.

## Methods

### Clinical specimen collection

Five patients diagnosed with cholangiocarcinoma in the Eastern Hepatobiliary Surgery Hospital were incorporated into the current study, including two iCCA, two GBC and one dCCA patients. Of note, none of the patients have undergone any preoperative treatments (chemotherapy, radiation, or antitumor medicines) prior to tumor resection. Tumor primary locus and metastasis as well as lymph node were consistently harvested for each participant, if available. Meanwhile, peripheral blood samples were collected prior to surgical procedures using anticoagulant tubes. The collection of human samples was approved by the Ethics Committee of Eastern Hepatobiliary Surgery Hospital (Shanghai, China).

### Tissue dissociation and processing

Fresh surgical resected samples were rinsed with Hank’s balanced salt solution (HBSS), which were subsequently minced and digested with digestive buffer, followed by incubation for 60 min at 37 °C with gentle shaking. The digestive buffer was configured by dissolving digestive enzyme into RPMI with 10% serum, including deoxyribonuclease type I, collagenase IV, and hyaluronidase type V. After digestion, the mixture was filtered using 300-mesh filter screen, and was then collected in the centrifuge tubes. Thereafter, leukocytes were isolated from tissues via density-gradient centrifugation in line with the manufacturer’s instructions. Filtered cell suspensions were centrifuged with 450 g for 8 min, and the resuspended precipitates were subsequently centrifuged with 50 g for 1 min. Washed cell suspensions were carefully layered on the surface of lymphoprep liquid, followed by centrifugation of 450 g for 25 min at room temperature. Tissue-derived leukocytes were obtained by collecting the middle layer of the mixture.

### Peripheral blood mononuclear cell isolation

Peripheral blood mononuclear cells (PBMCs) were isolated from blood sample using density-gradient centrifugation. Whole blood was collected by sodium heparin blood collection tubes and diluted three times with PBS (without calcium or magnesium). Suspensions were then superimposed on the surface of lymphoprep carefully, followed by centrifugation with 800 g for 15 min. Interphase-containing PBMCs were obtained and washed with PBS twice prior to further processing.

### Single-cell RNA-seq and TCR clonotype profiling

CountessII Automated Cell Counter (Thermo Fisher Scientific, USA) was applied to determine viability and density of mononuclear cells from tumor, lymph node, and peripheral blood. Single-cell suspensions were then processed for single-cell gene expression (scRNA-seq) as well as single-cell T cell receptor (TCR) clonotypes (scTCR-seq) using Chromium Controller. Single cells were adjusted to an ideal concentration of 5 × 10^5^ to 1 × 10^6^ cells/mL with a viability higher than 70%. Single-cell library was prepared in line with the protocol of 10X Genomics for Single Cell V(D)J and 5′ Gene Expression (10X Genomics, Pleasanton, CA, USA). In brief, single-cell suspensions were then loaded onto the Chromium Controller combined with the reverse transcription (RT) master mix and Gel beads containing barcode information. Following single-cell gel bead-in-emulsions reverse transcription, PCR amplification was carried out to generate adequate cDNAs for the construction of scRNA-seq as well as TCR V(D)J libraries. Thereafter, the libraries were sequenced on HiSeq4000 (Illumina) following the manufacturer’s specification.

### Single-cell RNA-seq processing

Cell Ranger software (Version 2.0) was applied to convert Illumina base call files to FASTQ files with the ‘cellranger mkfastq’ command, which were subsequently aligned to the hg19 human genome. Meanwhile, Cell Ranger carried out default quality control, and generate files containing raw unique molecular identifier (UMI) counts matrix. Downstream analyses were performed using the “Seurat” package (3.1.1) form R software (4.0.3). Cells with lower than 200 identified genes were removed during quality control process. Of note, quality-control parameters also included UMI counts, proportion of UMIs derived from mitochondrial and dissociation or sorting associated genes. After exclusion of low-quality cells, UMI counts were normalized by the function ‘NormalizeData’, in which normalization method was set as ‘logNormalize’ with the scale factor of 10000 total UMIs per cell. Thereafter, ‘FindVariableGenes’ function was performed with default parameters to detect 2000 highly variable genes, in which ribosome as well as heat shock response related genes were regressed out due to potential confounding effect. Principal component analysis (PCA) was subsequently conducted based on the processed expression matrix containing highly variable genes, followed by secondary UMAP visualization on selected PCA using the ‘RunUMAP’ function at a perplexity value of 30. Differentially expressed genes across clusters were calculated and identified using function “FindAllMarkers”.

Similarly, TCR sequencing data was generated and extracted via Cell Ranger. By obtaining positions 80–130 bp located in the hypervariable region, sequencing coverage of TCR molecules was normally high. Correspondingly, low coverage UMIs were filtered, which might be correlated with error and contaminations. Thereafter, filtered reads were mapped to the reference sequences via IgBlast (ftp://ftp.ncbi.nlm.nih.gov/blast/executables/igblast/release/1.7.0/) and then categorized in line with the TCR sequence represented them best. TCR sequence data were matched to Sc-seq data by using cell barcodes. Validating analysis revealed that obtained TCRs were never overlapped for cells from disparate participants. TCR sequence extracted form scTCR-seq can be employed as clonal identifier for T lymphocytes. T cells sharing consistent TCR were deemed to be derived from the identical clonal cells.

### Cell cycle and Trajectory analysis

Based on the relative expression levels of G2/M and S phase-related gene sets, cells were initially assigned a fraction of cycle. For the cells not expressed these cell cycle-related genes, it may be at G1 phase. ‘CellCycleScoring’ function was applied to generate cell cycle score which was then matched into the metadata. Meanwhile, the predicted classification for each cell in disparate proliferative phases was also calculated and grouped via the above package.

Trajectory as well as pseudotime analysis were performed using ‘Monocle’ algorithm that designed to identify differentially expressed genes (DEGs) varied across disparate clusters. Generalized additive models (GAMs) is established to determine the average expression level of each isoform. The formula was presented as follow:

$$g\left( {E\left( Y \right)} \right) = \beta 0^ + f1\left( {x1} \right)^ + f2\left( {x2} \right)^ + \cdots ^ + fm\left( {xm} \right),$$

where the *Y* and *xi*’s represents the response variable and predictor variables, respectively. The function *g* and function *fi*’s is link function and nonparametric functions, respectively. To estimate the relative gene expression level across cells, Tobit model was used accordingly. Therefore, the following formula of monocle’s GAM was adopted:

$$E\left( Y \right) = s\left( {\Psi t\left( {bx,si} \right)} \right)^ + \in ,$$

where Ψ*t* (*bx, si*) is the allocated pseudotime of cell and ϵ is the error term normally distributed around mean of zero. Function s is a cubic smoothing function with 3 effective degrees of freedom.

### Multiplex immunohistochemistry staining

iCCA tissue from 18 patients were stained using Opal Multiplex Immunohistochemistry Detection Kit (Akoya), followed by imaging with Vectra 3.0 Pathology Imaging System Microscope (Perkin-Elmer). Tissue slides were initially deparaffinized and rehydrated, followed by antigen retrieved via microwave treatment. Thereafter, slides were then treated with 3% H_2_O_2_ for 15 min and blocked with 1% BSA containing 0.1% Triton X-100 (Sigma). Slides were subsequently incubated with primary antibody, including anti-CD3 (Abcam cat#ab16669, dilution 1/100), anti-CD8 (Abcam cat# ab210067, dilution 1/150), anti-PD-1 (Abcam Cat# ab52587, dilution 1/50) and anti-XBP-1 (Abcam Cat# ab109221, dilution 1/200). Detection dye for each antibody were listed as follow: Opal570 dye (CD3), Opal520 dye (CD8), N700 dye (PD-1), Opal620 dye (XBP1). Meanwhile, DAPI was employed to counterstain nuclei. The digital images were analyzed using Halo Image Analysis software (indicalabs) with Highplex FL module. Immune cells were evaluated as the positive cell number in independent fields.

### CD8^+^ T Cell Isolation and In vitro cell culture

PBMCs were isolated from healthy individuals using Ficoll density-gradient centrifugation in accordance with the manufactory’s instruction. Thereafter, human CD8^+^ T cell positive selection kit (StemCell Technologies) was applied to enrich CD8^+^ T cells. CD8^+^ T cell were then stimulated with plate-bound anti-CD3 and anti-CD28 (Thermo Fisher Scientific) at a 1:1 ratio in 96-well plates for 72 h prior to subsequent experiments. On the day of harvesting, activated CD8^+^ T cells were resuspended with 50% diafiltrated supernatant from HIBEpiC, HCCC-9810 or GBC-SD, and cultured for additional 48 h. To testify the efficacy of IRE1α-XBP1 inhibition on T cells, 10 µM 4μ8C (EMD Millipore) was added to the RPMI medium for 24 h before harvesting.

HIBEpiC, HCCC-9810, and GBC-SD were purchased from the Shanghai Cell Bank of the Chinese Academy of Sciences, China. These cell lines were routinely cultured in complete RPMI 1640 medium containing 10% fetal bovine serum (FBS), penicillin (100 IU/ml), and streptomycin (100 µg/ml). Cells were maintained in the incubator at 37 °C and 5% CO_2_.

### Western blot analysis

Cells were collected and lysed with lysis buffer, followed by incubation on ice for 30 min. Lysates were subsequently centrifuged at 12,000 rpm for 30 min at 4 °C, prior to water bath for 5 min at 95 °C after mixing with SDS-loading buffer. Equal amounts of qualified samples were loaded onto and separated by 8–12% SDS polyacrylamide gel electrophoresis (Pulilai Co., Beijing, China), transferred to nitrocellulose membrane (Merk Millipore, Darmstadt, Germany). Thereafter, the transferred membrane was incubated with primary antibodies (IRE1α: Cell Signaling Technology, #3294, dilution 1/1000; XBP1: Cell Signaling Technology, #40435, dilution 1/1000; Beta Actin: proteintech, 66009-1-lg, dilution 1/1000) at 4 °C overnight, followed by incubation of HRP-conjugated anti-rabbit secondary antibody (proteintech, SA00001-2, dilution 1/5000) at RT for 1 h. The blots were visualized and analyzed by using the ECL system to determine the relative expressions (Fig. S1).

### Flow cytometry

After processing cells into single-cell suspensions, flow cytometry analysis was performed using antibodies purchased from BioLegend (CD279, #367410, dilution 1/100; TIGIT, #372710, dilution 1/100; CD8, #344714, dilution 1/100; XBP1, #647506, dilution 1/50). For the detection of surface markers, Cells were stained in PBS containing 2% FBS with fluorochrome-conjugated antibodies for 30 min at 4 °C. To determine the expression of intracellular cytokines, cells were permeabilized and fixed using the FoxP3 staining buffer kit (Thermo Fisher Scientific) in line with the manufacturer’s protocols. Aforementioned cytometric analyses were carried out through an LSR II instrument (BD Biosciences). All data were analyzed via FlowJo software (Version 10).

### Confocal microscopy

The Morphological alterations of ER were measured by applying Laser scanning confocal microscopy (LCSM). Collected cells were fixed with 4% paraformaldehyde for 20 min at 4 °C after PBS washes for 3 times, followed by permeabilization using block buffer (0.3% Triton X-100 in PBS) for 20 min at room temperature. After 3 times PBS wash, cells were then blocked with 1% bovine serum albumin (BSA) for 1 h at room temperature. Thereafter, Cells were stained with ER tracker (1:2000; Invitrogen, E34250) for 1 h in 5% CO2, 37 °C incubator after PBS washes for 3 times. Finally, after 3 times PBS wash, cells were stained with 4’, 6’-diamidino-2-phenylindole (DAPI; Sigma-Aldrich, D9542), and mounted onto slides. The slides were further observed through a laser scanning confocal microscope (Leica, Mannheim, Germany).

### Statistical analysis

All statistical analyses were conducted using R software (4.0.3) as well as GraphPad Prism software 7. All grouped data were shown as mean ± SEM. An unpaired student *t* test and one-way analysis of variance (ANOVA) were applied to assess statistical significance when two groups and more than three groups were compared, respectively. Two-tailed *P* values less than 0.05 were regard as statistical significance.

### Ethics approval and consent to participate

All procedures performed in studies involving human participants were in accordance with the Helsinki declaration. And all patients whose tissue samples were used in this research provided written informed consent, and the study protocol was approved by the committee for the Ethical Review of Research, Eastern Hepatobiliary Surgery Hospital.

### Reporting summary

Further information on research design is available in the Nature Research Reporting Summary linked to this article.

## Supplementary information

REPORTING SUMMARY

supplemental material

Supp Data

## Data Availability

Processed single-cell RNA sequencing data, T cell receptor sequencing data, and raw data are publicly available in Gene Expression Omnibus (https://www.ncbi.nlm.nih.gov/geo/info/linking.html.) under the accession number (GSE201425).

## Code availability

Our scRNA-seq analysis pipelines and codes can be obtained from the original author’s open source R package and script on Bioconducter (https://satijalab.org/seurat/; http://cole-trapnell-lab.github.io/monocle-release/docs/#getting-started-with-monocle; https://www.cellphonedb.org; https://www.bioconductor.org/packages/release/bioc/html/clonotypeR.html). The default standard parameters are used in the analysis process.
