# Supplementary material for: Single-cell atlas of diverse immune populations in the advanced biliary tract cancer microenvironment
Source: NPJ Precis Oncol. 2022 Aug 18;6:58. doi: 10.1038/s41698-022-00300-9 (PMC9388673; doi:10.1038/s41698-022-00300-9)
Supplement: Supplementary file 1 — REPORTING SUMMARY [file 41698_2022_300_MOESM1_ESM.pdf]

nature portfolio

Corresponding author(s): Hongyang Wang, Xiaoqing Jiang, Lei Chen

Last updated by author(s): Apr 6, 2022

Reporting Summary

Nature Portfolio wishes to improve the reproducibility of the work that we publish. This form provides structure for consistency and transparency in reporting. For further information on Nature Portfolio policies, see our Editorial Policies and the Editorial Policy Checklist.

Statistics

For all statistical analyses, confirm that the following items are present in the figure legend, table legend, main text, or Methods section.

n/a	Confirmed	
☐	☒	The exact sample size (n) for each experimental group/condition, given as a discrete number and unit of measurement
☐	☒	A statement on whether measurements were taken from distinct samples or whether the same sample was measured repeatedly
☐	☒	The statistical test(s) used AND whether they are one- or two-sided<br>*Only common tests should be described solely by name; describe more complex techniques in the Methods section.*
☐	☒	A description of all covariates tested
☐	☒	A description of any assumptions or corrections, such as tests of normality and adjustment for multiple comparisons
☐	☒	A full description of the statistical parameters including central tendency (e.g. means) or other basic estimates (e.g. regression coefficient) AND variation (e.g. standard deviation) or associated estimates of uncertainty (e.g. confidence intervals)
☐	☒	For null hypothesis testing, the test statistic (e.g. F, t, r) with confidence intervals, effect sizes, degrees of freedom and P value noted<br>*Give P values as exact values whenever suitable.*
☐	☒	For Bayesian analysis, information on the choice of priors and Markov chain Monte Carlo settings
☐	☒	For hierarchical and complex designs, identification of the appropriate level for tests and full reporting of outcomes
☐	☒	Estimates of effect sizes (e.g. Cohen's d, Pearson's r), indicating how they were calculated

Our web collection on statistics for biologists contains articles on many of the points above.

Software and code

Policy information about availability of computer code

Data collection	ScRNA-seq and TCR-seq data were collected by HiSeq4000 (Illumina). Data of Multiplex immunohistochemistry (mIHC) were collected by Polaris (PerlinElmer). Data of Flow Cytometry were collected by an LSR II instrument (BD Biosciences). The Morphological alterations of endoplasmic reticulum (ER) were observed through a laser scanning confocal microscope (Leica, Mannheim, Germany).
Data analysis	Cell Ranger software (Version 2.0); the "Seurat" package (3.1.1); R software (4.0.3); 'NormalizeData'; 'logNormalize'; 'FindVariableGenes'; Principal component analysis (PCA) ; 'RunUMAP'; 'FindAllMarkers'; 'CellCycleScoring' ; 'Monocle' algorithm with the default standard parameters were used for ScRNA-seq data analysis. Data of mIHC were analyzed with Halo Image Analysis software (indicalabs) with Highplex FL module. Data of Flow Cytometry were analyzed via FlowJo software (Version 10). All statistical analyses were conducted using R software (4.0.3) as well as GraphPad Prism software 7.

For manuscripts utilizing custom algorithms or software that are central to the research but not yet described in published literature, software must be made available to editors and reviewers. We strongly encourage code deposition in a community repository (e.g. GitHub). See the Nature Portfolio guidelines for submitting code & software for further information.

Data

Policy information about availability of data

All manuscripts must include a data availability statement. This statement should provide the following information, where applicable:

- Accession codes, unique identifiers, or web links for publicly available datasets
- A description of any restrictions on data availability
- For clinical datasets or third party data, please ensure that the statement adheres to our policy

Processed single-cell RNA sequencing data and raw data are publicly available in Gene Expression Omnibus (https://www.ncbi.nlm.nih.gov/geo/) under the accession number (GSE201425).

Field-specific reporting

Please select the one below that is the best fit for your research. If you are not sure, read the appropriate sections before making your selection.

☒ Life sciences ☐ Behavioural & social sciences ☐ Ecological, evolutionary & environmental sciences

For a reference copy of the document with all sections, see nature.com/documents/nr-reporting-summary-flat.pdf

Life sciences study design

All studies must disclose on these points even when the disclosure is negative.

Sample size	For ScRNA-seq and mIHC cohort, they were observational studies, thus no statistical methods used to predetermine to sample size. Two individual repeats were conducted for in vitro experiments.
Data exclusions	No data were excluded from the analyses.
Replication	Multiplex immunohistochemistry (mIHC) staining based on CD3, CD8, PDCD1, and XBP1 antibodies at the protein level in an additional 20 iCCA patients who received anti-PD-1 therapy and had available clinical response data defined by RECIST criteria (n=10, partial response (PR)/ complete response (CR); n=10, progressive disease (PD); tableS2) was used to further validate the ScRNA-seq results.<br>In vitro experiments were performed on two different cell lines(HCCC-9810 and GBC-SD) with at least 2 biologically independent replicates.
Randomization	Not relevant to our study because this was an observational study.
Blinding	mIHC analysis and in vitro experiments was performed in a blinded manner. For data analyses of ScRNA-seq, the statistical analysis was conducted automatically by the software.

Reporting for specific materials, systems and methods

We require information from authors about some types of materials, experimental systems and methods used in many studies. Here, indicate whether each material, system or method listed is relevant to your study. If you are not sure if a list item applies to your research, read the appropriate section before selecting a response.

Materials & experimental systems

n/a	Involved in the study	
☐	☒	Antibodies
☐	☒	Eukaryotic cell lines
☒	☐	Palaeontology and archaeology
☒	☐	Animals and other organisms
☐	☒	Human research participants
☒	☐	Clinical data
☒	☐	Dual use research of concern

Methods

n/a	Involved in the study	
☒	☐	ChIP-seq
☐	☒	Flow cytometry
☒	☐	MRI-based neuroimaging

Antibodies

Antibodies used	Antibodies Used for Multiplex Immunohistochemistry<br>Anti-Human CD3 Abcam Cat# ab16669<br>Anti-Human CD8 alpha Abcam Cat# ab210067<br>Anti-Human CD3 Abcam Cat# ab16669<br>Anti-Human PD1 Abcam Cat# ab52587<br>Anti-XBP1 Abcam Cat# ab109221<br>Antibodies Used for Flow Cytometry

	Anti-Human PD-1 BioLegend Cat# 329935<br>Anti-XBP-1s BioLegend Cat# 647505<br>Anti-Human TIGIT BioLegend Cat# 372722
Validation	Validation data of antibodies can be found on the suppliers' website.

Eukaryotic cell lines

Policy information about cell lines

Cell line source(s)	HIBEpiC, HCCC-9810 and GBC-SD were purchased from the Shanghai Cell Bank of the Chinese Academy of Sciences, China.
Authentication	The three cell lines were not authenticated.
Mycoplasma contamination	Cells were tested for mycoplasma as regularly as possible using PCR methods, and no mycoplasma contamination were detected.
Commonly misidentified lines<br>(See ICLAC register)	No misidentified cell line was used in this study.

Human research participants

Policy information about studies involving human research participants

Population characteristics	Five patients diagnosed with cholangiocarcinoma in the Eastern Hepatobiliary Surgery Hospital were incorporated into the current study, including two intrahepatic cholangiocarcinoma (iCCA), two gallbladder carcinoma (GBC) and one dCCA patients.<br>the validation cohort comprised 20 iCCA patients who underwent surgery and received anti-PD-1 therapy.<br>More details can be found in supplementary tables.
Recruitment	considering the accessibility of specimens from patients with advanced diseases, we pooled the three subtypes of BTC by necessity to obtain adequate sample size. Although these three different tumor types belong to biliary tract cancer (BTC), they have distinct genetic aberrations, clinical presentations and therapeutic approaches, which inevitably introduced bias.
Ethics oversight	the collection of human samples was approved by the Ethics Committee of Eastern Hepatobiliary Surgery Hospital (Shanghai, China).

Note that full information on the approval of the study protocol must also be provided in the manuscript.

Flow Cytometry

Plots

Confirm that:

☒ The axis labels state the marker and fluorochrome used (e.g. CD4-FITC).

☒ The axis scales are clearly visible. Include numbers along axes only for bottom left plot of group (a 'group' is an analysis of identical markers).

☒ All plots are contour plots with outliers or pseudocolor plots.

☒ A numerical value for number of cells or percentage (with statistics) is provided.

Methodology

Sample preparation	PBMCs were isolated from healthy individuals using Ficoll density-gradient centrifugation in accordance with manufactory's instruction. Thereafter, human CD8+ T cell positive selection kit (StemCell Technologies) was applied to enrich CD8+ T cells. CD8+ T cell were then stimulated with plate-bound anti-CD3 and anti-CD28 (Thermo Fisher Scientific) at a 1:1 ratio in 96-well plates for 72 hours prior to subsequent experiments. On the day of harvesting, activated CD8+ T cells were resuspended with 50% diafiltrated supernatant from HIBEpiC, HCCC-9810 or GBC-SD, and cultured for additional 48 hours prior to flow cytometry analysis.
Instrument	LSR II instrument (BD Biosciences)
Software	FlowJo software (Version 10)
Cell population abundance	Human CD8+ T cell positive selection kit (StemCell Technologies) was applied to enrich CD8+ T cells.
Gating strategy	Live CD8+ cells were gated based on FSC-A/SSC-A, in which we gated TIGIT+, PD-1 or XBP1+ cells for determining its percentage.

☐ Tick this box to confirm that a figure exemplifying the gating strategy is provided in the Supplementary Information.
