# Supplementary material for: Single-cell atlas of diverse immune populations in the advanced biliary tract cancer microenvironment
Source: NPJ Precis Oncol. 2022 Aug 18;6:58. doi: 10.1038/s41698-022-00300-9 (PMC9388673; doi:10.1038/s41698-022-00300-9)
Supplement: Supplementary file 2 — supplemental material [file 41698_2022_300_MOESM2_ESM.pdf]

Supplementary Material

Supplementary Table 1: sample information for single-cell RNA sequencing (scRNA-seq)

Patient ID	Clinical Information								Analysis	Sequencing Statistics			
	Age/Sex	Stage(TNM)	Tissue	TBIL (umol/L)	HBsAg	ALT (U/L)	CA199 (U/ml)	WBC ($*10^9/L$)		Estimated Number of Cells	Mean Reads Per Cell	Median Genes Per Cell	Sequencing Saturation
iCCA1	70/M	IV(T3N0M1)	primary focus	9.9	negative	46	122	8.8	scRNA-seq	4165	72780	831	85.3%
			peritoneal metastasis						scRNA-seq	4536	69754	1544	77.4%
			peripheral blood						scRNA-seq	5813	51976	1332	83.8%
iCCA2	52/M	IV(T3N0M1)	primary focus	12.5	negative	9	38.8	3.48	scRNA-seq	4936	79406	1683	85.3%
			liver metastasis						scRNA-seq	3847	110256	1514	87.7%
			peripheral blood						scRNA-seq	5105	56471	1282	88.9%
GBC1	54/F	IIIB(T3N1M0)	primary focus	184	negative	150	197	6.35	scRNA-seq	8364	45112	1136	68.1%
			liver invasion						scRNA-seq	5805	62172	1176	87.0%
			lymph node						scRNA-seq	5461	65034	1162	83.4%
			peripheral blood						scRNA-seq	6757	52026	1163	90.9%
GBC2	36/M	IIIB(T3N1M0)	primary focus	10.6	negative	24	26.3	6.23	scRNA-seq	6215	62292	1332	82.8%
			lymph node						scRNA-seq	3270	106442	1311	88.0%
			peripheral blood						scRNA-seq	5543	63456	1363	89.9%
dCCA	61/F	IIB(T3N0M0)	primary focus	62.1	negative	70	<2.0	5.33	scRNA-seq	4147	69618	1045	83.9%
			lymph node						scRNA-seq	4738	55824	1228	86.0%
			peripheral blood						scRNA-seq	5488	52464	1342	87.6%

abbreviation：iCCA: intrahepatic cholangiocarcinoma; GBC: gallbladder carcinoma; dCCA: distal cholangiocarcinoma; TNM: Tumor-Node-Metastasis; TBIL: total bilirubin; HBsAg: hepatitis B surface antigen; ALT: alanine amiotransferase; CA199: carbohydrate antigen 199; WBC: white blood cell.

Supplementary Table 2: sample information for multiplex Immunohistochemistry (mIHC)

patient ID	clinical information							analysis	Cell count				
	age/sex	clinical response	HbsAg	TBIL (umol/L)	WBC ($*10^{9}$/L)	CA199 (U/L)	ALT (U/L)		number of $PD1^{+}CD8^{+}$ T cells	number of $XPB1^{+}CD8^{+}$ T cells	number of Tex	number of $CD8^{+}$ T cells	number of $CD3^{+}$ T cells
iCCA01	28/M	PD	N	8.9	7.83	76.9	65	mIHC	0	767	854	1324	8654
iCCA02	51/M	PD	N	13.9	8.29	411.8	15	mIHC	34	1485	2411	3270	11801
iCCA03	53/M	PD	N	10.3	7.04	9.9	52	mIHC	49	202	287	867	2595
iCCA04	55/F	PD	N	9.7	6.21	42	41	mIHC	5	30	36	285	613
iCCA05	73/M	PD	N	17	7.53	261	16	mIHC	4	228	247	607	4046
iCCA06	49/F	PD	P	9.4	5.01	241	38	mIHC	118	5511	7535	9288	37233
iCCA07	51/M	PD	P	10.8	7.69	30.5	42	mIHC	7	7	26	54	79
iCCA08	55/F	PD	N	5.5	6.85	152	36	mIHC	125	8894	9521	15667	73599
iCCA09	46/F	PD	P	7.5	3.49	240	46	mIHC	1544	1139	5157	8336	19760
iCCA10	52/M	PR	P	13.9	9.22	49.2	33	mIHC	1859	895	3917	5145	9122
iCCA11	44/M	PR	N	13.4	8.98	2	66	mIHC	755	2288	6394	9026	16754
iCCA12	49/M	PR	N	22.9	6.29	16.9	240	mIHC	3308	283	4811	6770	14861
iCCA13	47/F	PR	P	10.2	6	21.9	25	mIHC	437	2375	3629	4822	12905
iCCA14	63/F	PR	N	14.4	6.78	>1000	23	mIHC	169	200	479	854	1736
iCCA15	63/F	PR	N	25.8	3.92	149	19	mIHC	354	137	690	2357	7432
iCCA16	72/F	PR	N	302	5.31	>1000	111	mIHC	9	131	144	818	2674
iCCA17	53/F	PR	N	13.4	4.22	>1000	20	mIHC	9	34	43	2143	5463
iCCA18	68/M	CR	N	11.4	9.03	0.6	62	mIHC	49	193	350	1588	6647

Abbreviations: M: male; F: female; PD: Progressive disease; PR: Partial response; CR: Complete response; iCCA: intrahepatic cholangiocarcinoma; HBsAg: hepatitis B surface antigen; N: negative; P: positive; TIBL: total bilirubin; WBC: white blood cell; CA199: carbohydrate antigen 199; ALT: alanine aminotransferase; Tex: exhausted $CD8^{+}$T cells.

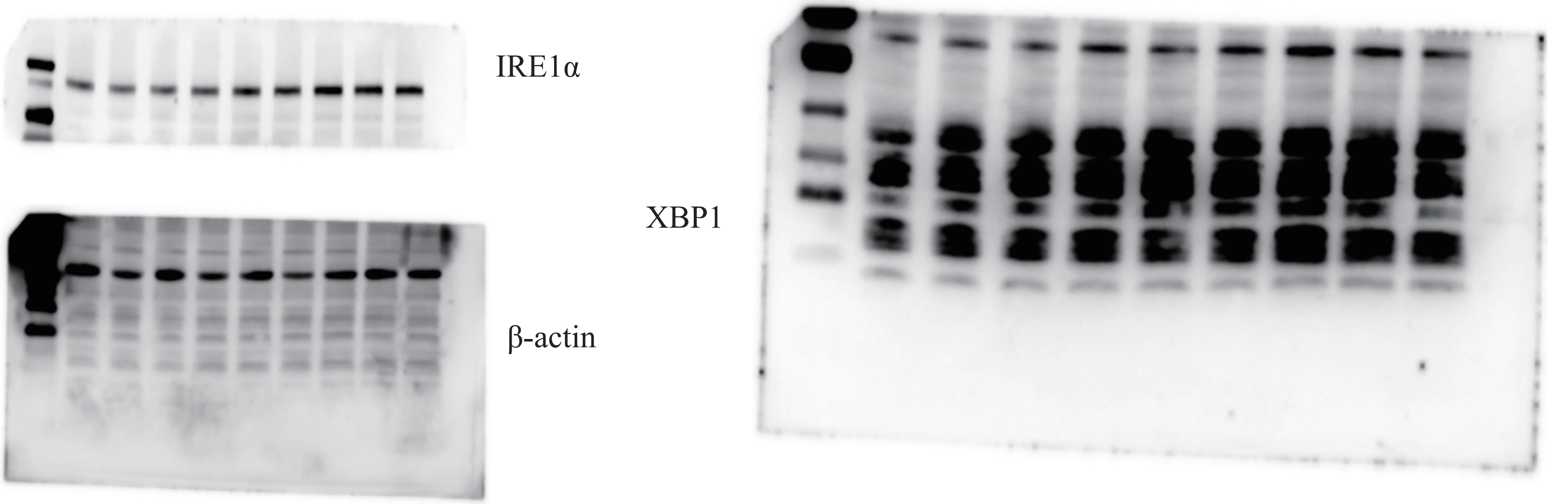

Supplementary Figure 1. Uncropped Blots of Figure 6c

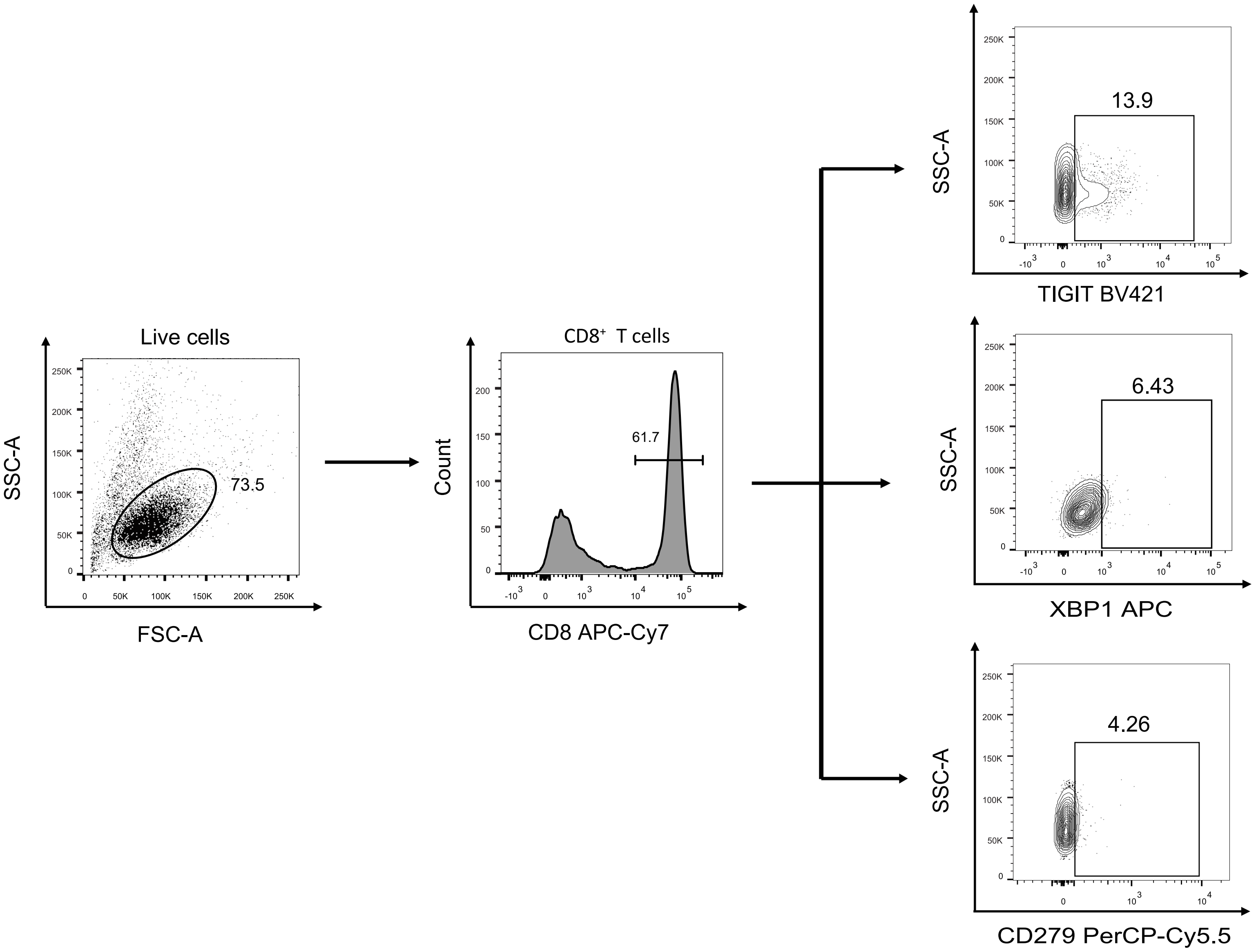

Supplementary Figure 2. Flow cytometry gating strategy for Figure 6d, 6e and 6f.
